# Tailoring chiral optical properties by femtosecond laser direct writing in silica

**DOI:** 10.1038/s41377-023-01080-y

**Published:** 2023-02-20

**Authors:** Jiafeng Lu, Jing Tian, Bertrand Poumellec, Enrique Garcia-Caurel, Razvigor Ossikovski, Xianglong Zeng, Matthieu Lancry

**Affiliations:** 1 Institut de Chimie Moléculaire et des Matériaux d’Orsay, Université Paris Saclay, Orsay, 91405 France; 2 Key Laboratory of Specialty Fiber Optics and Optical Access Networks, Joint International Research Laboratory of Specialty Fiber Optics and Advanced Communication, Shanghai Institute for Advanced Communication and Data Science, Shanghai University, Shanghai, 200444 China; 3 LPICM, CNRS, Ecole Polytechnique, Institut Polytechnique de Paris, Palaiseau, 91128 France

**Keywords:** Ultrafast photonics, Laser material processing

## Abstract

An object that possesses chirality, that is, having its mirror image not overlayed on itself by rotation and translation, can provide a different optical response to a left- or right-handed circular polarized light. Chiral nanostructures may exhibit polarization-selective optical properties that can be controlled for micro-to-nano optical element engineering. An attractive way to induce such complex nanostructures in three-dimension in glass is femtosecond laser direct writing. However, the mechanism of femtosecond laser induced chirality remains to be unveiled due to complex physical and chemical processes occurring during the ultrashort light-matter interaction. Here, a phenomenological model is proposed and is built on two-layers phase shifters to account for this laser-induced optical chirality in an initially achiral material (silica glass). This model is based on the observation that femtosecond laser induced nanogratings own two principal contributions to its aggregate birefringent response: a form and a stress-related one. By refining this formalism, a multilayer approach is developed to imprint on demand optical rotation. Values up to +/-60° at 550 nm within an optimal 80 μm thickness in silica glass are possible, corresponding to the highest value in a glass to date. These results provide new insights of circular-optical control in micro-nano optical manufacturing and open new opportunities for photonics applications.

## Abstract (web summary)

A two-layers model is proposed to reveal that femtosecond laser direct writing chirality inside silica glass has two contributions: a form birefringence and a stress related birefringence.

## Graphical abstract

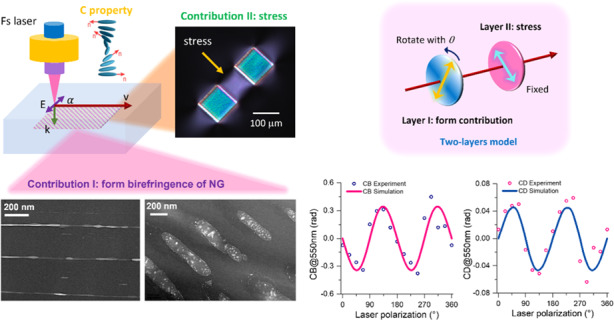

## Introduction

From fundamental physics of light-matter interaction to fabrication of targeted optical properties in highly complex optical engineering, femtosecond (fs) laser plays a key role in laser manufacturing. Ultrashort light pulses can precisely deposit light energy in a given transparent material volume by controllable focusing conditions. Nonlinear absorption of high-density photon energy leads to the creation of free electrons plasma within few fs, that is before the electron-phonon energy transfer to the lattice (typ. >10 ps in silica). Thus, at low repetition rate, the glass network heating is decoupled from light exposure and from the plasma itself, which enables localized modifications or even breakdown without surrounding damage. Such nonlinear processes contribute to multiple types of modifications according to laser parameters: (1) an isotropic average index variation (Type I); (2) anisotropic subwavelength nanogratings yielding to high form birefringence (Type II); (3) micro/nano voids due to localized micro-explosions (Type III)^1^. To date, these fs laser-induced modifications have found many applications in most branches of nonlinear science, ranging from plasma physics and nano-photonics to material science, bio-photonics and quantum information science^2,3^.

Recent progress highlights that a fs laser beam (axially symmetric intensity distribution, linear polarized with orthogonal incidence) can create optical chirality inside an achiral material through a laser direct writing within Type II regime^4–6^. This concept shed light on a new approach for tailoring chiral optical properties in three dimensional (3D) providing a wider landscape of laser manufacturing. A prerequisite to exploit such new potentials is to elucidate the mechanism how one can manipulate these chiral optical properties. As a first model Poumellec et al.^6^ suggested that the breaking of symmetry arises from a volume torque due to the combined action of a stress field and a direct current (DC) electric field. This torque is the source of the chirality originating from pulse front tilt (PFT), focusing conditions and laser polarization. The misalignment between DC electric field $\vec E_{DC}$ and the material polarization field $\vec P$ generates a volume torque $\vec P\Lambda \vec E_{DC}$. Its orientation and sign are controllable and may twist the matter to create chiral atomic arrangements. Although the quenched glass network records evidence of a part of molecular activities during the light-matter interaction, the origin of this optical chirality imprinting remains unknown. There is no information to support if the observed circular properties and related optical rotation originate from a laser-induced molecular optical activity such as for sucrose or α-quartz with its chiral lattice arrangement i.e., a so-called intrinsic chirality.

However, if we transfer the concept from molecular activity to optical fundamentals, it can lead to another plausible explanation, that is, circular optical properties can originate from several internal linear birefringence and dichroism contributions with non-parallel, non-perpendicular eigen axes^7^ i.e., an extrinsic chirality^8^. Typically, fs laser irradiation in silica glass can result in a Type II modification with nanograting structures and associated mechanical stress at appropriate laser parameters. These nanogratings mainly exhibit an obvious structural anisotropy resulting in a strong uniaxial negative form birefringence^9^. The origin of this anisotropic optical property lies on the subwavelength regular arrangement of nanolayers with a refractive index contrast. It is the specific morphology of nanogratings firstly carried out by Shimotsuma et al. in 2003 that leads to the exciting development of both physical research and practical applications of fs laser induced nanogratings^10^. In addition, irradiation of silica glass in this regime results to a net volume expansion^11^, which correlates with the formation of porous nanolayers inside the nanogratings^12,13^. This, in turn, creates a significant birefringence due to the photoelastic tensor of glass. Several teams^14–16^ have highlighted the presence of such stress fields within both Type I and Type II regimes. Following this view, we propose here that fs laser induced circular optical properties could originate from the concomitant contributions of a form birefringence and a stress-induced birefringence.

In this paper we tentatively “disassemble” the dependence of the form and stress contributions with respect to the laser polarization direction. In a simple view, the slow/fast axis of the form birefringence is controlled by the laser polarization direction and the related retardance amplitude by the laser fluence. In addition, the polarization dependence of the stress-induced birefringence is investigated through a simple approach based on stress-engineered waveplates as described in previous works^15,17^. A two-layers model is then developed based on Mueller formalism to quantitatively explain the creation of both linear and circular anisotropic optical properties. Finally, we exploit this model to engineer chiral optical properties following two different designs, namely multilayer “nanogratings based waveplates” and “stress-engineered waveplates”. The following results provide the evidence that the origin of fs laser induced circular properties includes two contributions and propose a strategy for tailoring chiral optical properties in any glasses by fs laser direct writing.

## Results

### Writing polarization dependence of fs laser induced chiral optical properties

**Fig. 1:**
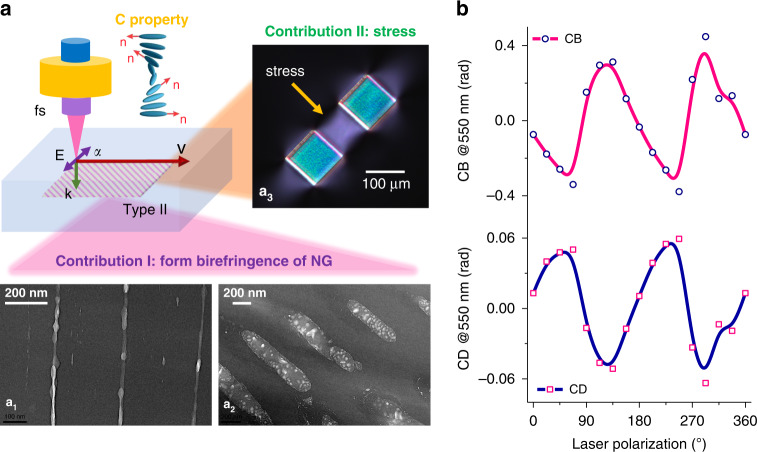
Fs laser induced circular properties and their contributions. **a** Conceptual scheme of fs laser inscription of nanogratings possessing circular properties with two contributions; TEM images of nanogratings with cleaving perpendicular (a_1_) and parallel (a_2_) to the laser propagation direction; (a_3_) crossed polarizers microscope image of a stress-engineered waveplate between two irradiated zones (called stress bars). **b** CB (upper) and CD (below) evolutions according to laser polarization. Measurements are done at 550 nm

The proposed concept that the Type II nanostructures written by fs laser consists of two contributions (form birefringence and stress birefringence) is schematically shown in Fig. 1a. In the following it is further experimentally demonstrated by fs laser direct writing homogeneous squares made of a set of lines inside bulk silica glass, using appropriate laser parameters within the Type II processing window (more details can be found in “Materials and methods section”).

To characterize the overall anisotropic optical properties after fs irradiation, one can use Mueller spectro-polarimetry in transmission mode and related definitions namely: linear birefringence (LB), 45°-linear birefringence (LB’), total linear birefringence (TLB), linear dichroism (LD), 45°-linear dichroism (LD’), total linear dichroism (TLD), circular birefringence (CB), and circular dichroism (CD). Since, it is more practical in polarimetry, all these properties are described as linear (or circular) phase difference in this paper. All the definitions, descriptions and units are depicted in Table 1. In addition, since CB will effectively rotate the plane of polarization, we can define an optical rotation $\theta _{{{{\boldsymbol{rot}}}}}$ in degrees. Note that optical activity is mostly used for describing intrinsic chirality but CB and optical rotation are more general and thus more appropriate for this paper. Finally, CD is defined as the difference of absorptions between left- and right-handed circular polarized beams and can result in an elliptical light polarization.

**Table 1:** Linear and circular optical properties

Property	Definition	Commonly described in polarimetry	Unit
LB (linear birefringence)	${\it{\Delta }}{{{\boldsymbol{n}}}}_{{{\boldsymbol{L}}}} = \left( {{{{\boldsymbol{n}}}}_{{{\boldsymbol{x}}}} - {{{\boldsymbol{n}}}}_{{{\boldsymbol{y}}}}} \right)$	${{{\boldsymbol{LB}}}} = \frac{{2\pi }}{\lambda }\left( {{{{\boldsymbol{n}}}}_{{{\boldsymbol{x}}}} - {{{\boldsymbol{n}}}}_{{{\boldsymbol{y}}}}} \right) \cdot {{{\boldsymbol{d}}}}$	radians
LB’ (45°-linear birefringence)	${\it{\Delta }}{{{\boldsymbol{n}}}}_{{{{\boldsymbol{L}}}}^\prime } = \left( {{{{\boldsymbol{n}}}}_{45^\circ } - {{{\boldsymbol{n}}}}_{ - 45^\circ }} \right)$	${{{\boldsymbol{LB}}}}^\prime = \frac{{2\pi }}{\lambda }\left( {{{{\boldsymbol{n}}}}_{45^\circ } - {{{\boldsymbol{n}}}}_{ - 45^\circ }} \right) \cdot {{{\boldsymbol{d}}}}$	radians
TLB (total linear birefringence)	${\it{\Delta }}{{{\boldsymbol{n}}}}_{{{{\boldsymbol{TLB}}}}} = \left( {{{{\boldsymbol{n}}}}_{{{\boldsymbol{e}}}} - {{{\boldsymbol{n}}}}_{{{\boldsymbol{o}}}}} \right)$ < *for a uniaxial material* >	${{{\boldsymbol{TLB}}}} = \sqrt {{{{\boldsymbol{LB}}}}^2 + {{{\boldsymbol{LB}}}}^{\prime 2}}$	radians
TLB fast axis angle	*Lower refractive index axis*	$\theta _{{{\boldsymbol{B}}}} = \frac{1}{2} \cdot {{{\boldsymbol{arctan}}}}\left( {\frac{{{{{\boldsymbol{LB}}}}}}{{{{{\boldsymbol{LB}}}}^\prime }}} \right)$	degrees
LD (linear dichroism)	${\it{\Delta }}\kappa _{{{\boldsymbol{L}}}} = \left( {\kappa _{{{\boldsymbol{x}}}} - \kappa _{{{\boldsymbol{y}}}}} \right)$	${{{\boldsymbol{LD}}}} = \frac{{2\pi }}{\lambda }\left( {\kappa _{{{\boldsymbol{x}}}} - \kappa _{{{\boldsymbol{y}}}}} \right) \cdot {{{\boldsymbol{d}}}}$	radians
LD’ (45°-linear dichroism)	${\it{\Delta }}\kappa _{{{{\boldsymbol{L}}}}^\prime } = \left( {\kappa _{45^\circ } - \kappa _{ - 45^\circ }} \right)$	${{{\boldsymbol{LD}}}}^\prime = \frac{{2\pi }}{\lambda }\left( {\kappa _{45^\circ } - \kappa _{ - 45^\circ }} \right) \cdot {{{\boldsymbol{d}}}}$	radians
TLD (total linear dichroism)	${\it{\Delta }}\kappa _{{{{\boldsymbol{TLD}}}}} = \left( {\kappa _{{{{\boldsymbol{high}}}}} - \kappa _{{{{\boldsymbol{low}}}}}} \right)$	${{{\boldsymbol{TLD}}}} = \sqrt {{{{\boldsymbol{LD}}}}^2 + {{{\boldsymbol{LD}}}}^{\prime 2}}$	radians
TLD fast axis angle	*Lower attenuation axis*	$\theta _{{{\boldsymbol{D}}}} = \frac{1}{2} \cdot {{{\boldsymbol{arctan}}}}\left( {\frac{{{{{\boldsymbol{LD}}}}}}{{{{{\boldsymbol{LD}}}}^\prime }}} \right)$	degrees
CB (circular birefringence)	${\it{\Delta }}{{{\boldsymbol{n}}}}_{{{\boldsymbol{C}}}} = \left( {{{{\boldsymbol{n}}}}_ - - {{{\boldsymbol{n}}}}_ + } \right)$	${{{\boldsymbol{CB}}}} = \frac{{2\pi }}{\lambda }\left( {{{{\boldsymbol{n}}}}_ - - {{{\boldsymbol{n}}}}_ + } \right) \cdot {{{\boldsymbol{d}}}}$	radians
Optical rotation	_/_	$\theta _{{{{\boldsymbol{rot}}}}} = \frac{\pi }{\lambda }\left( {{{{\boldsymbol{n}}}}_ - - {{{\boldsymbol{n}}}}_ + } \right) \cdot {{{\boldsymbol{d}}}} \cdot \frac{{180^\circ }}{\pi }$	degrees
CD (circular dichroism)	${\it{\Delta }}\kappa _{{{\boldsymbol{C}}}} = \left( {\kappa _ - - \kappa _ + } \right)$	${{{\boldsymbol{CD}}}} = \frac{{2\pi }}{\lambda }\left( {\kappa _ - - \kappa _ + } \right) \cdot {{{\boldsymbol{d}}}}$	radians

${{{\boldsymbol{n}}}}_{[]}$: refractive index; $\kappa _{[]}$: absorption index; ***d***: thickness of anisotropic layer; ***λ***: probe light wavelength; *x*: *x* axis; *y*: *y* axis; *e*: extraordinary light; *o*: ordinary light; “-”: left-handed; “+”: right-handed

Transmission electron microscope (TEM) analysis confirm that nanogratings are formed by the alternation of “uniform” and nanoporous glass layers, as depicted in Fig. 1a_1_ and Fig. 1a_2_. The nanoporous areas represent the nanoplanes that have been cleaved in their plane, the areas between them appearing black are areas that, although having been irradiated, have remained dense^18^. The net volume expansion, induced by the formation of these nanopores, creates a strong compressive stress field in and around the irradiated zone. Such effect is clearly visible from Fig. 1a_3_.

To explore the underpinning mechanisms yielding to chiral optical properties and their relationships with laser parameters, the effect of laser polarization dependence is investigated firstly. Experimentally, a set of different laser polarization directions are adopted to imprint a set of milimeter size homogeneous samples with keeping fixed all other parameters. Figure 1b exhibits the laser polarization dependence of CB and CD of the fs laser-irradiated SiO_2_ samples, measured at the probe light wavelength of 550 nm. The maximum CB (or CD) can be observed from X + 45° writing configuration (linear polarization azimuth ***α*** = 45°, 135°, 225° and 315°). However, there is almost no CB (or CD) when the polarization is along ***α*** = 0°, 90°, 180° and 270°. In addition, anisotropic circular properties exhibit a non-symmetric angular response with a 180° periodic dependence at different azimuth ***α*** (with respect to the scanning direction that was chosen along x-axis i.e., 0°).

### Writing polarization dependence of the form birefringence, LB_form_

**Fig. 2:**
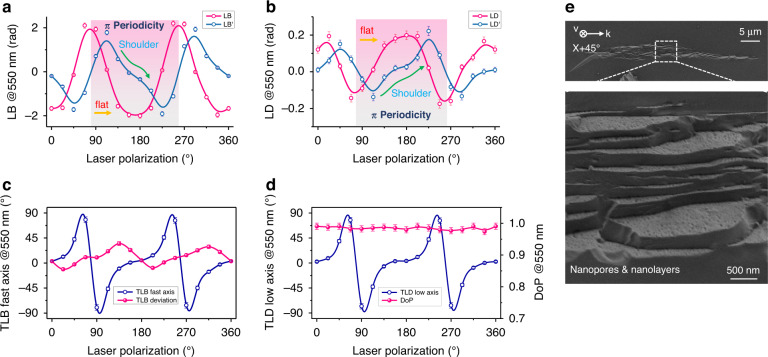
Laser polarization dependence of anisotropic properties of fs laser irradiated SiO_2_ sample. **a** LB and LB’ evolutions according to laser polarization. **b** LD and LD’ evolutions according to laser polarization. **c** TLB fast axis evolution and its deviation (its difference compared to laser polarization *α*, −90°< deviation ≤ 90°). **d** TLD “low attenuation axis” evolution and the degree of polarization (DoP). **e** SEM images of fs laser irradiated region using a X + 45° writing configuration. Measurements are done at the wavelength of 550 nm

Fs laser written nanogratings exhibit a linear birefringence that is strongly related to the laser polarization^9^. Note that if the samples are solely made of a birefringent layer whose orientation is linearly dependent of the laser polarization (that is, “ideal nanogratings” with neither tilt nor stress effect on their orientation), both LB and LB’ functions will be perfectly periodic with a 45° shift with respect to each other. It is worth noting that when neutral axis of the birefringence is parallel/perpendicular to x (or y) axis, the LB comes to be 0 but LB’ reaches maximum whereas the TLB remains almost constant. However, as shown in Fig. 2a, a LB deviation is observed from an ideal sinusoidal shape, exhibiting a “flat zone” around 180°. Following the same trend, LB’ owns a periodic modulo 180° evolution, but its angular response also deviates from sinusoidal shape and presents a “shoulder” around 180°±22°. Besides, the LD and LD’ show similar results as shown in Fig. 2b.

To figure out the inner mechanism of these irregularities with respect to the linear polarization azimuth, both TLB fast axis and its deviation (its difference compared to ***α***) are displayed in Fig. 2c. Similar results, from the TLD “low attenuation axis” are shown in Fig. 2d. For completeness, the degree of polarization (DoP) measured at 550 nm is included and appears larger than 98% revealing a quite negligible depolarization in the investigated spectral range. Finally Fig. 2e shows typical SEM (scanning electron microscope) micrographs of the fs laser track (written nanogratings) using a X + 45° configuration. This configuration gives the opportunity to observe not only regularly spaced nanolayers but also their intrinsic nanoporous nature in a single image. The laser track appears quite homogeneous, and its typical length is around 40 μm.

### Writing polarization independence of the stress-induced birefringence, LB_stress_

For crystalline materials amorphized by fs laser irradiation, it is possible to observe a guiding zone in the surrounding region having undergone compressive mechanical stresses all around the irradiated zone, which exhibits a volume expansion^11^. Similar results have been demonstrated in silica glass within the Type II regime (i.e., nanogratings formation)^14^. When a stress load is applied to a material, the latter develops a linear birefringence according to the photoelastic effect. To perform stress profile measurements, Champion et al. have used fs laser-written specific design and derived an equation to model the stress field accordingly^11^. Their results indicate that the amplitude of the compressive stress should depend on the nanolayers orientation with respect to the writing direction^14^ but not the stress orientation^18^.

**Fig. 3:**
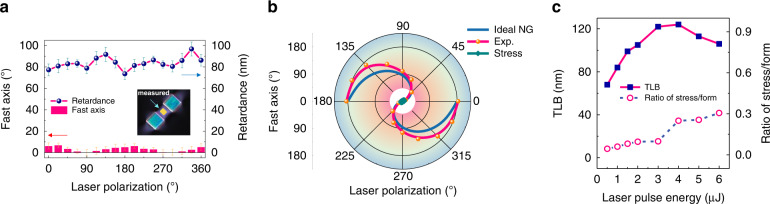
Laser polarization independence of stress-induced birefringence. **a** LB_stress_ fast axis orientation and retardance according to the laser polarization direction; inset refers to the crossed polarizers microscope image of the stress bars (in blue) creating a pure stress zone with the mark of the measured area (the 0° reference is the direction of the written lines i.e., along x axis). **b** TLB fast axis direction evolutions of “ideal nanogratings” (blue), measured fs laser-induced nanogratings (pink) and pure stress (green) versus laser polarization direction. **c** Evolutions of TLB and LB_stress_/LB_form_ ratio at different laser pulse energies. All measurements are done at 550 nm

Thus, to experimentally investigate such effects we designed a set of dedicated samples (see experimental details in “Materials and methods section”). This is exemplified in Fig. 1a_3_ inset where the crossed polarizers image reveals the formation of birefringence not only within the two irradiated areas (blue interference color) but also in-between these two stress bars and around which is attributed to mechanical stress^15^. The measurements of both its related retardance and fast axis to the laser polarization azimuth ***α*** are shown in Fig. 3a. We could confirm that the fast/slow axis direction is primarily determined by the writing geometry and does not depend on the laser writing polarization as a first approximation (excepted at the edges of the written lines where the slow axis is azimuthally distributed^14^). Thus, for single lines or superposition of lines, there is a quasi-uniaxial loading along the line and the fast axis direction of the stress-induced birefringence is parallel to the lines for volume expansion^11^. In other words, whether the laser writing polarization is aligned along x or y, the fast/slow axis orientation of the stress-induced birefringence remains unchanged. However, there exists a slight perturbation of the retardance function that indicates a variation of the birefringence amplitude within 20% in agreement with ref. ^14^. For completeness, the fast axis orientation is found to deviate by a maximum of 8° from the line direction especially when the nanolayers are perpendicular to the scanning direction. This deviation could be caused by a non-uniform stress field distribution across the sample thickness^17^ since there is no compensation for spherical aberration.

Figure 3b summarizes evolutions, as a function of laser polarization, of (1) the fast axis for “ideal nanogratings” (a form birefringence with nanolayers perpendicular to laser polarization), (2) experimental Type II nanogratings, and (3) a pure stress zone. The stress related data are close to the center of the polar plot, which indicates a laser polarization independence of LB_stress_ orientation as discussed above. The measured fast axis of the nanogratings possesses an obvious deviation compared to the theory for “ideal nanogratings”. Furthermore, we have tentatively decoupled the LB_form_ contribution from the one coming from stress. The measured TLB and LB_stress_/LB_form_ ratio according to the laser pulse energy are shown in Fig. 3c. Here, as a simplified approach, “X + 45°” configuration is used to ensure that the measured LB mostly originates from stress-induced birefringence whereas the measured LB’ comes from the form birefringence. It is worth pointing out that LB_stress_ contributes from 5% up to 30% when increasing the energy. Within the experimental conditions (1030 nm, 250 fs, 1.5 μJ/pulse), LB_stress_ contributes around 10% to the overall birefringence TLB, which is in good agreement with the thermal stability of TLB^15^ (also reproduced for comparison in Fig. 5e).

### A simple but powerful two-layers quantitative model

#### Overview of the model

**Fig. 4:**
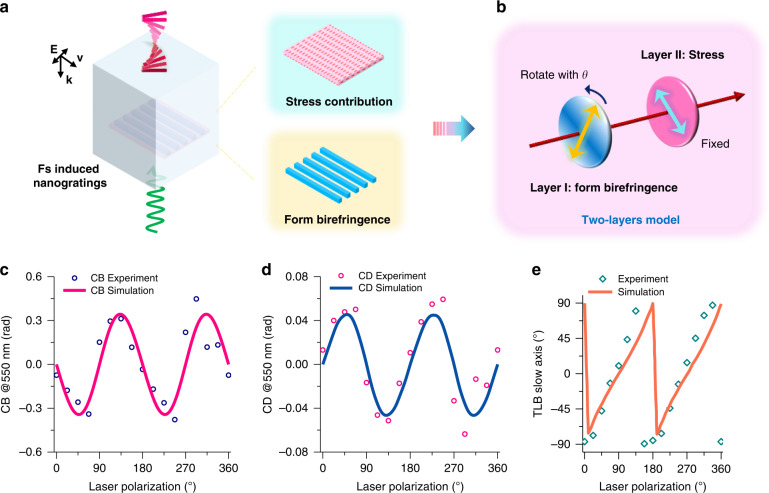
The two-layers model and the simulation results. **a** The schematic of the two contributions that lead to the laser induced circular optical properties. **b** The schematic of two-layers model consists of two linear retarders. Simulated evolutions according to laser polarization compared with experimental results for CB (**c**), CD (**d**) and TLB slow axis (**e**). Calculations and measurements are done at 550 nm

In this section, a two-layers model is proposed to reveal the inner mechanism of the fs laser-induced circular optical properties as shown in Fig. 4a. To obtain a robust understanding of the circular optical properties imprinting, this model is constructed in the Mueller formalism instead of Jones matrix, because (1) the light is partially polarized after passing through the laser irradiated area and (2) it is more suitable when all properties are coexisting. The main hypothesis is built on the basis of the propagation of light in inhomogeneous media composed of anisotropic linear layers. In a simple view, every “interaction volume” in the irradiated area is considered to have two linear contributions, which are finally “sumed” through Mueller formalism.

In the following, we consider the case of two layers with linear anisotropic optical properties, and whose neutral axis orientations may be different, as depicted in Fig. 4b. This fulfills the minimum condition to create an object exhibiting both linear and circular properties (not entangled or entangled depending on the type of decomposition applied). As a direct consequence, the CB would originate from several (at least 2) internal LB contributions with both non-parallel and non-perpendicular neutral axes^7^. Similarly, CD band would result from the cumulative action of LD and LB present in respectively two different layers, also having non-parallel and non-perpendicular neutral axes. Following this model, the amplitude of the observed CD and CB will not only be related to the LD or LB optical phase shift of each contribution, but also to the misalignment between their respective neutral axes.

#### Mathematical description

It is recalled that the anisotopic properties are associated with a potentially attenuating phase shifter (dichroic) and therefore contain a linear birefringence term LB and a linear dichroism term LD, both expressed in radians unit. In the Mueller formalism chosen here, the optical phase shifter MDR (a linear retarder) is written according to the following relation:

1 $${{{\boldsymbol{MDR}}}}\left( {{{{\boldsymbol{R}}}},{{{\boldsymbol{P}}}}_{{{{\boldsymbol{si}}}}}} \right) = \left[ {\begin{array}{*{20}{c}} 1 & { - {{{\mathbf{cos}}}}(2{{{\boldsymbol{P}}}}_{{{{\boldsymbol{si}}}}})} & 0 & 0 \\ { - {{{\mathbf{cos}}}}(2{{{\boldsymbol{P}}}}_{{{{\boldsymbol{si}}}}})} & 1 & 0 & 0 \\ 0 & 0 & {{{{\mathbf{sin}}}}(2{{{\boldsymbol{P}}}}_{{{{\boldsymbol{si}}}}}) \cdot {{{\mathbf{cos}}}}({{{\boldsymbol{R}}}})} & {{{{\mathbf{sin}}}}(2{{{\boldsymbol{P}}}}_{{{{\boldsymbol{si}}}}}) \cdot {{{\mathbf{sin}}}}({{{\boldsymbol{R}}}})} \\ 0 & 0 & { - {{{\mathbf{sin}}}}(2{{{\boldsymbol{P}}}}_{{{{\boldsymbol{si}}}}}) \cdot {{{\mathbf{sin}}}}({{{\boldsymbol{R}}}})} & {{{{\mathbf{sin}}}}(2{{{\boldsymbol{P}}}}_{{{{\boldsymbol{si}}}}}) \cdot {{{\mathbf{cos}}}}({{{\boldsymbol{R}}}})} \end{array}} \right]$$

where *R* is the linear phase shift (corresponds to TLB when expressed in radians, here we assume the fast axis along to 0° so that TLB is equal to LB) and *P*_*si*_ translates the di-attenuation (or dichroism) property of the phase shifter (corresponds to TLD when expressed in radians). Note that a zero linear dichroism corresponds to a value of ${{{\boldsymbol{P}}}}_{{{{\boldsymbol{si}}}}} = \pi /4$. The angle $\theta _{1,2}$ is defined as the angle between the fast axis of the layer (layer 1 and layer 2, respectively) and the x-axis of the benchmark of the laboratory (defined by the laser compressor plane). After determining the Mueller matrix *M* corresponding to the assembly of these two phase shifters as shown in Eq. (2), the resulting matrix can be formally decomposed by exploiting the logarithmic approach developped by Ossikovski et al.^19^. Here, ${{{\boldsymbol{M}}}}_{{{\boldsymbol{R}}}}$ represents the rotation matrix. The effective anisotropic optical properties then can be extracted from the modeled medium.

2 $$\begin{array}{ll}{{{\boldsymbol{M}}}} = \left[ {{{{\boldsymbol{M}}}}_{{{\boldsymbol{R}}}}\left( {\theta _2} \right) \cdot {{{\boldsymbol{MDR}}}}\left( {2^{{{{\boldsymbol{nd}}}}}\;{{{\boldsymbol{layer}}}}} \right) \cdot {{{\boldsymbol{M}}}}_{{{\boldsymbol{R}}}}\left( { - \theta _2} \right)} \right]\\ \qquad\cdot\, \left[ {{{{\boldsymbol{M}}}}_{{{\boldsymbol{R}}}}\left( {\theta _1} \right) \cdot {{{\boldsymbol{MDR}}}}\left( {1^{{{{\boldsymbol{st}}}}}\;{{{\boldsymbol{layer}}}}} \right) \cdot {{{\boldsymbol{M}}}}_{{{\boldsymbol{R}}}}\left( { - \theta _1} \right)} \right]\end{array}$$

#### Physical origin of the two layers

Correspondingly, one of the two phase shifters will be representative of the form birefringence attributed to ideal nanogratings (i.e., no tilt, no stress effect on their orientation) and thus exhibits a strong uniaxial negative linear birefringence as reported by Bricchi et al. ^9^ and a non-zero “apparent” linear dichroism related to anisotropic light scattering. Note that LD is attributed to nanopores formation and their arrangement in nanolayers (positive for Xx writing, negative for Xy writing) so it is not directly related to the birefrigence through a Kramers-Kronig relationship. As the angle between the laser polarization and the writing direction is changing during the writing process (different laser polarization configurations), the direction $\theta _1$ of the fast axis has to be varied accordingly. The second phase shifter is attributed to the presence of a stress-induced birefringence (but no LD) that will guide us in the definition of its properties, of which the amplitude is lower (typically an order of magnitude) than the first layer. This model may seem very simplistic, however, it retains the essential physics to represent the results because it takes into account the more likely sources of birefringence: LB_form_ and LB_stress_, as shown in Fig. 4b.

#### Exploiting the two-layers model

Quantatively, the value of the parameters $\left( {{{{\boldsymbol{R}}}},{{{\boldsymbol{P}}}}_{{{{\boldsymbol{si}}}}}} \right)$ adopted were adjusted on our experimental results. Generally, the first layer representing the form contribution of nanogratings is assigned at $- \pi /2$ for the parameter *R*_*1*_ related to the TLB i.e., a delay of $\lambda /2$ with a fast axis aligned along x (for example 275 nm at the wavelength of 550 nm) and 0.70 rad for the parameter ${{{\boldsymbol{P}}}}_{{{{\boldsymbol{si}}}}{1}}$ related to the TLD. Concerning the second layer, a typical value of 0.44 rad is found for the parameter *R*_*2*_ and $\pi /4$ for the parameter ${{{\boldsymbol{P}}}}_{{{{\boldsymbol{si}}}}{2}}$ corresponding to no dichroism. The first layer will rotate linearly with the ***α*** orientation of the laser polarization, that is, $\theta _1 = \alpha$. In contrast, the stress layer is proved to be laser polarization independent (see Fig. 3a). Therefore, the fast axis angle is parallel (respectively slow axis is perpendicular) to the laser scaning direction (here, it is along x axis) and thus $\theta _2 = 0^\circ$. Now the Mueller matrix of the model can be simplifed to be:

3 $${{{\boldsymbol{M}}}} = {{{\boldsymbol{MDR}}}}\left( {2^{{{{\boldsymbol{nd}}}}}\,{{{\boldsymbol{layer}}}}} \right) \cdot \left[ {{{{\boldsymbol{M}}}}_{{{\boldsymbol{R}}}}\left( {\theta _1} \right) \cdot {{{\boldsymbol{MDR}}}}\left( {1^{{{{\boldsymbol{st}}}}}\,{{{\boldsymbol{layer}}}}} \right) \cdot {{{\boldsymbol{M}}}}_{{{\boldsymbol{R}}}}\left( { - \theta _1} \right)} \right]$$

Consequently, CB and CD polarimetric results computed by employing the Mathcad^©^ software are shown in Fig. 4c and Fig. 4d, respectively. Obviously, the simulation accounts for the experimental results quite well. Apparently, CB and CD both exhibit a *π* period with the polarization azimuth *α* (with respect to the scanning direction i.e. along x axis). We demonstrate here that CB arises from a combined effect of the LB_form_ and LB_stress_ contributions with non-parallel and non-perpendicular eigen axes. In the meanwhile CD comes from a combined effect of the LD (co-existing with the LB_form_ in the 1^st^ layer) and the LB_stress_ with non-parallel and non-perpendicular eigen axes. Furthermore, CB and CD approach the maximum value when the angular misalignment between the two eigen axes is ±45°. Note that CB and CD amplitudes depend on the polarization orientation with respect to the scanning direction, which could be related to some “asymmetric orientational writing”^20^. In addition, the calculated TLB slow axis is directly related to the observed deviation of the TLB slow axis orientation that is not perfectly linearly dependent on the laser writing polarization azimuth angle, as depicted in Fig. 4e.

### Optical chirality engineering employing a multilayer configuration

From a practical point of view, the operating principle of the proposed two-layers model provides a powerful framework (e.g., multilayer configuration) towards circular optical properties engineering both in amplitude and sign. To demonstrate the feasibility of inscribing high chiral optical properties, we investigated multilayers-based designs built either on “nanogratings based waveplate” or on “stress-engineered waveplate”. For sake of comparison, we also developed a “two-layers LB_form_ waveplate” made from two-layers nanogratings subsequently annealed for 30 minutes at 1150 °C to relax the stress field.

#### CB engineering using “nanogratings based waveplate”

**Fig. 5:**
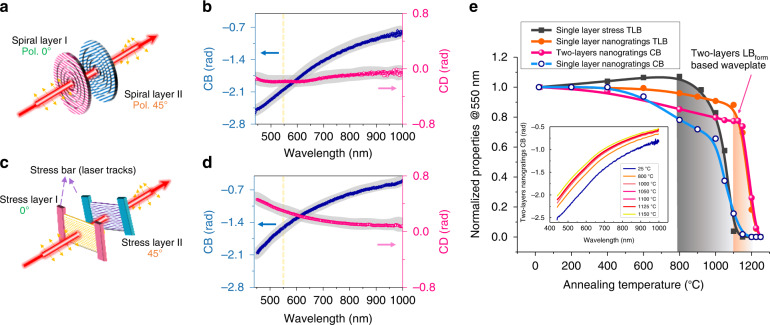
Circular anisotropic optical property engineering by exploiting a multilayer writing strategy. **a** Diagram of two layers of nanogratings, 45° misaligned. **b** Spectral circular properties of the “nanogratings based waveplate” within 450–1000 nm. **c** Diagram of two layers of stress, 45° misaligned. **d** Spectral circular properties of the “stress engineered waveplate” within 450–1000 nm. **e** Thermal stability of linear and circular properties. Measurements are done at 550 nm. Inset depicts the CB spectral evolution of two-layers “nanogratings based waveplate”. *Single layer stress TLB and single layer nanogratings TLB data are reproduced with the permission from ref*. ^15^

To obtain the maximum CB property, a misalignment of 45° between the two nanogratings layers should be used as shown in Fig. 5a. Figure 5b depicts the circular properties spectral dispersion extracted from Mueller spectro-polarimetry, which highlights a CB up to −2.5 rad measured at 450 nm, the highest value reported in a glass to the best of our knowledge. In comparison to our previous best result of −0.4 rad, the CB value of −2.1 rad (measured at 550 nm) is raised to more than 5 times. In principle, this CB value refers to an optical rotation of −60° within an optimal compactness of 80 μm, which is 20 times more than the optical rotation (calculated to be 2.7° at 550 nm for the same 80 μm thickness) in quartz^21^.

Here the spectral dependence of the circular properties originates from the linear ones^15^. Indeed, within the form birefringence model, the retardance $({{{\boldsymbol{n}}}}_{{{\boldsymbol{x}}}} - {{{\boldsymbol{n}}}}_{{{\boldsymbol{y}}}}) \cdot {{{\boldsymbol{d}}}}$ is relatively independent of the wavelength (***λ***) especially for long wavelengths ($\lambda > > \Lambda$, nanogratings period). However, when expressed in radians unit, the corresponding LB (or LD) increases at short wavelengths following the expression: ${{{\boldsymbol{LB}}}} = \frac{{2\pi }}{\lambda } \cdot ({{{\boldsymbol{n}}}}_{{{\boldsymbol{x}}}} - {{{\boldsymbol{n}}}}_{{{\boldsymbol{y}}}}) \cdot {{{\boldsymbol{d}}}}$ (rad). The same explanation typically stands for LB_stress_^15^. As a result, the CB amplitude that depends on (1) LB_form_, LB_stress_ amplitudes and (2) their angular misalignment increases at short wavelengths.

Towards further engineering applications, one should consider the spectral loss of the multilayered waveplates. For “nanogratings-based waveplate”, the main losses originate from the Rayleigh scattering due to the intrinsic nanoporous nature of nanogratings^5^. This light scattering owns a $1/\lambda ^4$ dependence leading to strong anisotropic losses in the UV-Vis range (transmittance >70% measured at 550 nm) but can be minimized by working within the Type X regime^22^ where transmittance can be higher than 95% at 550 nm.

#### CB engineering using “stress-engineered waveplate”

The “stress-engineered waveplate” exhibits only LB (with no LD) whose orientation is mostly dictated by the laser scanning geometry. Following this approach, we exploit fs laser direct writing to imprint stress bars (a set of lines) thus creating an irradiation-free stress zone in between^17^. Note that the birefringence orientation is manipulated by changing the direction of the stress bars arrangement. Experimentally, a 45° misalignment is employed between the two stress-engineered layers as shown in Fig. 5c. By measuring the Mueller matrix within the “irradiation-free aperture”, a high CB up to −2.1 rad can be measured at 450 nm as depicted in Fig. 5d. Similar to the previous design, we take the CB value of −1.57 rad measured at 550 nm into account for comparison. Quantitatively, one can easily create an optical rotation of +/−45° measured at 550 nm. In comparison to nanogratings, the absorption coefficient for the “stress engineered waveplates” reveals negligible absorption losses below 1 cm^−1^ over the whole spectral range from 200 nm up to 1600 nm (i.e., very close to the one of pristine SiO_2_)^15^, which is further strengthen this approach.

#### On the relative influence of stress and nanogratings

Furthermore, not only to fabricate a two-layers “LB_form_-based waveplate” but also to gain insights in the understanding of fs laser-induced circular properties, we perform step isochronal annealing. From previous results shown in Fig. 5e, stress-induced birefringence can nearly be erased after annealing temperature of 1100 °C (fully after 30 minutes at 1150 °C), which is accompanied by a full erasure of CB for a single nanogratings layer. On the contrary, the TLB only exhibits a decay of only 10% in the same annealing conditions. This confirms that the form birefringence contribution is much larger than the one from stress.

#### Now turning to the CB thermal stability

The two-layers “nanogratings based waveplate” depicts two principal features. First, from 400 °C to 1100 °C, the CB slightly decreased to around 80% of its initial value (prior annealing), which correlates with the stress relaxation. Above this temperature, a sort of two-layers “LB_form_ only” waveplate is obtained from which stress effects were released. Here the CB can still achieve −1.59 rad measured at 550 nm, which corresponds to an optical rotation of −45.6°. Secondly, above 1175 °C, the CB experiences a steep decay together with TLB that is attributed to nanopores erasure^18^. Therefore, this clearly indicates that (1) CB can effectively be achieved by non-parallel non-perpendicular assembly of “ideal nanogratings” without any stress assistance and (2) the major contribution to the measured CB is related to the nanogratings form birefringence.

## Discussion

From the previous sections, it has been demonstrated that, following a single-layer writing geometry, the fs laser-induced circular properties originate from the coexistence of (1) form birefringence and (2) stress-induced birefringence. Theoretically, circular birefringence can be observed, not only in chiral materials with a chiral molecular arrangement (called intrinsic chirality, like sugar or quartz that are optically active chiral media) but also in achiral materials that exhibit a chiral arrangement considering the probe light propagation direction (for instance, oblique incidence or chiral arrangement at larger scale, micrometric scale discussed in this paper). Specifically, in our model, the written object is a “chiral object” related to an extrinsic chirality. Indeed, the structure of two linear layers (LB_form_ and LB_stress_) that own non-parallel non-perpendicular slow axes cannot be superimposed by its mirror image. This chiral optical object shows a chiral optical arrangement of optical properties, which thus creates CB as confirmed by the model based on Mueller formalism.

### Effect of the form birefringence

The form birefringence of “ideal nanogratings” (no tilt of their orientation with respect to laser polarization) has its fast axis aligned with the laser polarization direction. At first, Kazansky et al. proposed that nanogratings are mainly caused by the interference of the laser field and bulk plasma wave, which was then revisited by different authors as a multiple scattered wave interference pattern that structures the free electrons plasma density^10^. Recently, it was revealed that nanogratings are formed by an ultrafast decomposition of silica i.e., nanopores formation inside these nanolayers^12^. Three cornerstones prove the existence of nanopores embedded in a step-by-step nanogratings formation process. First, the SEM investigation reported in 2013 (ref. ^13^) revealed the nanopores. Second, the TEM investigations presented herein (Fig. 1a_1_ and Fig. 1a_2_) provide a clear picture that nanolayers are made of an assembly of elongated nanopores. The formation of nanopores is attributed to SiO_2_ decomposition into ${{{\boldsymbol{SiO}}}}_{2(1 - {{{\boldsymbol{x}}}})} + {{{\boldsymbol{x}}}}\cdot {{{\boldsymbol{O}}}}_2$. This leads to an expulsion of ionized oxygen atoms from the lattice based on a tensile stress-assisted decomposition mechanism^23^ that was recently revisited as plasma mediated nanocavitation process^24^. The oxygen atoms are thus found in the interstitial position and recombine to form molecular oxygen. The typical diameter of nanopores ranges from 10 to 50 nm depending on the deposited energy^25^. Finally, the last cornerstone is attributed to small-angle X-ray scattering measurements that further confirmed the formation of elongated nanoporous structures within the nanolayers^26,27^. All the proposed theories and experimental results confirm that the nanogratings orientate ideally or theoretically perpendicular to the laser polarization. However, from our polarimetric data, the observed TLB angular deviation indicates either a presence of a nanogratings tilt and/or a presence of at least one more contribution to the TLB that is non-parallel non-perpendicular to the form birefringence^7^ e.g., the stress-induced birefringence.

### Effect of the PFT

The effect of PFT could be a possible contribution to the observed deviation creating a tilt in the nanolayers orientation as investigated by Dai et al. in 2014 (ref. ^28^). The polarization dependence arises due to the spatio-temporal properties of the ultrafast laser beam quantified by spatial chirp, PFT and angular dispersion^29,30^. The PFT produced by temporal and spatial chirps (e.g., due to a slight misalignment of the laser compressor) can lead to both the “quill writing” and the anisotropic photosensitivity phenomena^20,29,31,32^. However, the PFT is capable of interpreting only a small part of the deviation because the PFT-induced nanograting rotation achieves only a few degrees at a hundred fs/mm tilt^28^. Indeed, the experimental proof of neutral axis deviation in our results exhibits an amplitude up to 35°, which is far above the level that PFT can usually achieve. Moreover, note that the polarization dependence of this deviation matches quite well with the one of the circular properties. This deviation is thus likely related to the stress field.

### Effect of the stress field

Ultrashort laser pulses enable the formation of a “quasi-free” electron plasma as an intermediate step between light absorption and structural modifications in the glass network. In particular within the Type II regime, the plasma is structured under the influence of light in relation with the solid. The spatial structuring of the plasma density gives rise to a force field in the solid, which then leads, after extinction of the light pulse, to the imprinting of a stress field. More specifically, there are two possible processes:

(1) “Self-structuring” of the quasi-free electron plasma under the electromagnetic field creates a structured DC field. This DC field distorts the ionized atomic network (partly elastically and plastically). When the laser light is off and after electron relaxation, the elastic part that was balanced by the electromagnetic force disappears, but a plastic strain and a stress field still survive.

(2) The structured plasma imprint structural changes like point defects or nanovoids, the concentration of which follows the plasma density. This may correspond to a change of lattice density and thus to the development of a stress field. For example, in SiO_2_, this agrees with a reported net volume expansion^11^, which correlates with the formation of porous nanolayers^12,13^. Locally, the effective glass volume is expanded and induces a permanent strain field^12^_,_ and therefore a stress field appears within and around the laser-modified region. Depending on the laser exposure conditions (including polarization), the overall stress can be enhanced or minimized^14,33^ leading to tunable stress-induced birefringence values from 10^-5^ up to ~10^-3^ accordingly^34–36^. Note that such stress-induced birefringence influences the written anisotropic optical properties but also likely the nanogratings themselves^37^. Therefore, within the Type II regime^14,38–42^, the formation of nanogratings is not solely creating form birefringence but also a stress-induced birefringence contribution that participate to the measured total birefringence^38,43,44^.

### About TLB neutral axis deviation

The fast axis evolutions of these two contributions are summarized in Fig. 3b and compared to the one of nanogratings, revealing some deviations. Based on the above discussion we suggest that the significant deviation of the TLB fast axis is due to the stress birefringence that is in general non-parallel and non-perpendicular to the nanolayers themselves. This results in a rotation of TLB neutral axis whose amplitude increases with (1) ratio of stress birefringence to the form birefringence and (2) the misalignment between their respective neutral axes (the maximum being 45°).

### Towards chiral optical properties exploitation

Finally, the fs-induced anisotropic circular properties originate not solely from the form birefringence but from a “cooperation” of this contribution and the stress one, but how do they quantitatively contribute to the imprinted optical chirality? Firstly, from the annealing results shown in Fig. 5e, the CB of two-layers “nanogratings-based waveplate” is dominated by the LB_form_, which can be easily controlled by laser writing parameters (e.g., pulse energy or writing speed)^45^. To further answer this question, our two-layers model not only provides the quantitative meaning of fs laser-induced optical chirality but also can be expanded to other more complex situations. This includes multilayers contributing to diverse anisotropic properties, including circular properties by simply adjusting the layers 3D assembly and the parameters adopted in each layer. Furthermore, such a multilayer strategy opens the door to a new strategy in laser manufacturing optical devices that require circular optical properties and even circular properties without any linear ones. Note this is not a simple linear accumulation of single layers but a vectorial combination for high optical chirality construction. One could arrange different layers including pure LB_form_ (accompanied by annealing strategy), pure stress or their combination with diverse amplitudes and angular misalignment. Interestingly also, the circular optical property cannot only be accumulated but also be compensated by creating the circular property with reverse sign.

## Conclusion

In the light of the current state of the art, we quantitatively interpreted the fs laser-induced optical chirality observed and characterized in silica glass. This property is attributed to the co-existence of two phenomena: nanogratings anisotropic properties (linear form birefringence and dichroism) and a stress-induced linear birefringence. Besides, the misalignment between eigen axes of these two contributions results in a symmetry breaking (a so-called extrinsic chirality) and creates chiral optical properties (CB and CD). It is worth pointing out that one can tailor both the amplitude and sign of the circular properties by adjusting the optical design and laser parameters.

The fundamental interest in fs laser-induced nanostructures that possesses the intrinsic parameters (periodicity, nanoporosity, 3D rotation), allows an effective control of birefringence slow/fast axis and even inducing circular optical properties that lead to the engineering of unique integrated optical devices with 3D space variant birefringence, 3D geometric phase optics and more generally refractive index tensor profiling. This result brings further understanding of the vectorial nature of light-matter interaction and allows designing any kind of 3D structured light beam in terms of phase, amplitude and polarization.

In addition, this approach can be implemented with a multilayer strategy to achieve on-demand optical rotation between [−90°, 90°] and within less than 200 μm. This ability to manipulate optical rotation opens possibilities to optical property engineering of any given transparent material. For instance, developing birefringent devices operating in a broad spectral range—including achromatic components or passive optical isolators—would benefit to many fields such as photonics integrated circuits, polarimetry, spectroscopy, attosecond pulse generation, terahertz, military and domotics applications.

## Materials and methods

### Definitions

LB and LB’ are defined as the difference of refractive indices between two othogonal linear polarized beams with a dimentionless unit. However, it is more practical in polarimetry to use linear phase difference to describe the LB and LB’ properties, which is adopted in this paper. Because it shows the collective influence to the probe light manipulated by our samples. Hence TLB can be calculated by ${{{\boldsymbol{TLB}}}} = \sqrt {{{{\boldsymbol{LB}}}}^2 + {{{\boldsymbol{LB}}}}^{\prime 2}}$ in radians with its fast axis angle. CB is defined as the difference of refractive indices between left- and right-handed circular polarized beams with a dimensionless unit. Similarly, it is common using circular phase difference to describe the CB property. LD, LD’, TLD and CD refer to the difference of the absorption between two othogonal linear/circular polarized beams respectively and are treated similarly as birefringence ones, as shown in Table 1.

### Femtosecond laser set-up

The laser beam is delivered from a fs laser system operating at $\lambda = 1030\;nm$, ${{\Delta }}t_{sp} = 250\;fs$ and outputting at a repetition rate of 100 kHz with an average power up to 10 W (Amplitude Systèmes, Pessac, France). Aspheric lens of 0.6 numerical aperture (NA) and 0.16 NA are adopted for fabricating Type II structure and stress bars, respectively, with a focusing depth of 500 μm below the surface. Based on the preliminary calibrations, the laser parameters are settled with appropriate pulse energy and scanning speed falling into the Type II window (typically 1 mm s^-1^ writing speed at 1.5 μJ for getting a smooth writing) inside a 1-mm-thick silica glass (Suprasil CG, Heraeus, Hanau, Germany). According to the theory reported in ref. ^46^ and using the plasma absorption reported in ref. ^47^, i.e., around 15% at 1.5 μJ and 0.16 NA, we can estimate the intensity of our used fs laser beam to be 8.8 TW cm^-2^ (0.16 NA). Then it is difficult to give a reliable intensity at 0.6 NA due to the lack of absorption data, but it should be very close to the clamping intensity that has been estimated to be around 50-60 TW cm^-2^ in silica^48^.

### Laser writing configurations

We define the laser configuration as “scanning direction ***v*** + laser polarization ***α***”, for example, here X + 45° refers to the laser scanning direction is along the x axis and the laser polarization ***α*** = 45°, with a typical error smaller than 0.5°. For simplicity, X + 0° and X + 90° are often replaced by “Xx” and “Xy”. A wide range of writing configurations is investigated here. Homogeneous squares generally with a size of 1.5 mm × 1.5 mm are carried out by unidirectional line scanning with 1 μm increment (to avoid “quill writing” and diffraction effects), which are suitable for the Mueller matrix spectro-polarimetry. Towards investigating the laser polarization dependence, the laser linear polarization is oriented from 0° (along the x axis defined by the laser compressor plane taken as a reference) up to 360° with a step of 22.5°.

#### For “nanogratings-based waveplates”

Two uniform disks (one using 0° polarization, and another using 45° with fixed other parameters) are written using a spiral trajectory with a diameter of 1.5 mm and a 1 μm pitch. The laser track length is 40 μm in z-axis (depth) and we put 100 μm distance in-between two layers, which means the two layers are almost independent layers. In addition, since the stress decreases exponentially out of the laser tracks, we can neglect the influence of the stress induced by the first layer when writing the second one.

#### For “stress-engineered waveplates”

The stress bars consisting of two squares (or two rectangles) made of lines are written for creating a strong stress field in between. Each stress bar is made of 5 written layers with a 35 μm increment along z-axis for ensuring a continuous writing of thick stress bars. Then we write another two stress bars to create the second stress layer with a 45° rotation. The increment between two stress layers is 100 μm to ensure almost independent layers. More details can be found in our previous work^15^.

### TEM observations

The interior of the nanogratings has been examined by cleaving the sample after laser irradiation. If the nanograting planes are parallel to the cleavage, the interior structure could be observed with field emission electron microscope, which could reveal small features otherwise covered by conductive coating. Further, high-resolution transmission electron microscopy could be applied to analyze the internal structure of nanogratings through transversal and longitudinal views with FIB technique (see Fig. 1(a_1_) and Fig. 1(a_2_)). Here, we used commercial TOPCON 002B electron microscope (200 kV with a resolution of 0.18 nm). This gives a better understanding of the internal structure of nanoplanes generation and nanopores distribution in the irradiated region of the silica glass. The FIB technique (Zeiss Neon 60, current 50 pA, accelerating voltage 30 kV) is used to dissect nanogratings embedded in silica into small slices with a thickness of 50 nm without invasive nanoplanes in the modified region.

### Mueller matrix characteristics

Spectral normalized Mueller matrices are recorded with a spectroscopic Mueller ellipsometer (Smart SE, JY HORIBA) in transmission at normal incidence in the wavelength range $\lambda \in 450\!\!-\!\!1000\;nm$. Normally a Smart SE spectroscopic ellipsometer is used to measure the properties of thin films with a reflection mode. But here we used in fact a modified version of this ellipsometer to create a Mueller spectro-polarimeter as described in the literatures^49,50^. Specifically, a polarization state generator and a polarization state analyzer were added in such equipment to determine the whole Mueller matrices in transmission mode. The sample is probed with at least four (sometimes more) different input polarized states that are generally elliptical, this allows to determine the whole 16 elements of the Mueller matrix, including the mixed information of linear and circular anisotropic optical properties with their sign or orientation and also the degree of polarization. The samples are oriented in such a way that their writing/scanning axis is set horizontal (+/−1°) in the reference frame of the Mueller ellipsometer. To extract these properties, the Mueller matrices measured experimentally are then decomposed using the logarithmic decomposition method^51,52^ to tentatively “disentangle” the anisotropic optical properties. In that way the effective values of LB, LB’, LD, LD’, CB, and CD, can be treated independently. What has a physical meaning is the so-called TLB, which represents the effective birefringence seen by a light probe. For a complete view TLB has to be associated with its neutral axes angle (i.e., fast/slow axis in our uniaxial case). Typical error bars within investigating spectral range remains smaller than 1° for all these properties. Thus, TLB and TLD can be calculated as well as their neutral axis (typ. within +/−2°). Apart from the multi-spectral behavior, all anisotropic optical properties are then extracted at a fixed wavelength of 550 nm.

### Imaging and birefringence measurement

Optical images are measured using an Olympus BX51 polarizing optical microscope (Olympus, Tokyo, Japan) equipped with a “de Sénarmont” compensator. A “de Sénarmont” strategy employs a highly precise quarter-waveplate companied with a feasible 180-degree-rotation polarization analyzer to achieve the retardance measurement with an accuracy of few nanometers at 550 nm. Notice that in only crossed polarized configuration, the birefringence image has different colors due to different values of retardance according to Michel-Levy scale. For example, in Fig. 1(a_3_), the color of the “stress bars” is blue because of a relatively high retardance while the grey part between the stress bars refers to a lower retardance in the pure stress area. In addition, we have performed quantitative linear birefringence imaging measurements at 550 nm using “Open Polscope”. Note that, we use small size of samples (typically 0.1 mm × 0.1 mm) for these measurements since the equipment are microscopes themselves. Therefore, the overall cartography of the birefringence amplitude together with its slow axis orientation can be determined successfully.

### Simulation method

We define a two linear retarders model under Mathcad^®^ software. Separately, the parameters of LB and LD can be added into the two linear retarders with both amplitudes and signs. The fast axis of the first retarder (refers to form birefringence contribution) is varied from 0° up to 360°, which is corresponding to the laser polarization direction change. Differently, the fast axis of the second retarder (refers to the stress birefringence contribution) is fixed at 0° in agreement with the writing geometry i.e., a set of lines written along x axis.

## Data Availability

Data is available from corresponding author upon reasonable requirements.
